# Decomposition and oxidation of methionine and tryptophan following irradiation with a nonequilibrium plasma jet and applications for killing cancer cells

**DOI:** 10.1038/s41598-019-42959-4

**Published:** 2019-04-29

**Authors:** Giichiro Uchida, Yusuke Mino, Tensho Suzuki, Jun-ichiro Ikeda, Takashi Suzuki, Kosuke Takenaka, Yuichi Setsuhara

**Affiliations:** 1 Joining and Welding Research Institute, Osaka University, Ibaraki, Osaka, 567-0047 Japan; 2 Graduate School of Medicine, Osaka University, Suita, Osaka, 565-0871 Japan; 3 Analytical and Measuring Instruments Division, Shimadzu Corporation, Kyoto, Kyoto, 604-8511 Japan; 4 Present Address: Graduate School of Medicine, Chiba University, Chiba, 260-8670 Japan

**Keywords:** Biomedical engineering, Chemical engineering

## Abstract

We present evidence for the decomposition and oxidation of amino acids in aqueous solution following irradiation with a nonequilibrium plasma jet. Of 15 amino acids tested in cell culture medium, plasma irradiation induced a marked chemical change in methionine and tryptophan due to the effective production of reactive oxygen species by plasma-water interaction. We also report that plasma-treated methionine and tryptophan aqueous solutions can kill cancer cells, greatly decreasing the viability of human endometrial carcinoma (HEC-1) cancer cells due to the presence of decomposition or oxidation products generated from the amino acid. Plasma-treated methionine and tryptophan aqueous solutions also induced an anti-cancer effect on cancer-initiating cells.

## Introduction

Atmospheric nonequilibrium plasma jets are of interest for biomedical applications such as wound disinfection and the treatment of cancer tumors^1–4^. Atmospheric nonequilibrium plasmas have the unique characteristics of a relatively high electron temperature of 2–4 eV and a low gas temperature under atmospheric pressure conditions^5–7^. High-energy electrons can produce chemically rich gas-phase environments containing reactive oxygen species (ROS) at room temperature in the open air. The beneficial therapeutic effects of atmospheric nonequilibrium plasmas are generally attributed to excited oxygen species in the air. Biomedical applications of nonequilibrium plasma jets require the generation of ROS in an aqueous solution^8–14^, thereby allowing significant modification of the characteristics of biomaterials and the activation of cells by ROS in aqueous solution.

The application of nonequilibrium plasmas to cancer therapy has been reported by many research groups^15–20^. An indirect plasma treatment protocol was recently used for a cell viability assay: first, the liquid was irradiated using an atmospheric plasma, then the plasma-treated liquid was added to cancer cells^21–32^. This indirect plasma treatment may be applicable to targeting cancer cells inside the body. Tanaka *et al*. reported that glioblastoma brain tumor cells are selectively killed when exposed to plasma-treated cell culture medium^22^. Conventional cell culture medium comprises more than 10 different amino acids, plus glucose, vitamins, and inorganic salts such as NaCl. Consequently, it is necessary to study the interaction between the plasma and these many components to understand the mechanism underlying the killing of cancer cells by plasma. In this study, on the other hand, we focus on amino acids because their total concentration in cell culture medium can be as high as 1000 mg/l and amino acids are predicted to be modified by plasma irradiation. Additionally, a basic study on the interaction between plasma and amino acids is important for other biomedical applications of plasmas because about 20% of a cell is composed of protein, which consists of 20 kinds of amino acids. Our measurements showed that some amino acids are oxidized or decompose upon plasma irradiation and that the observed antitumor effect is induced by products generated from these amino acids.

## Results

### Chemical changes in amino acids upon plasma treatment

**Figure 1:**
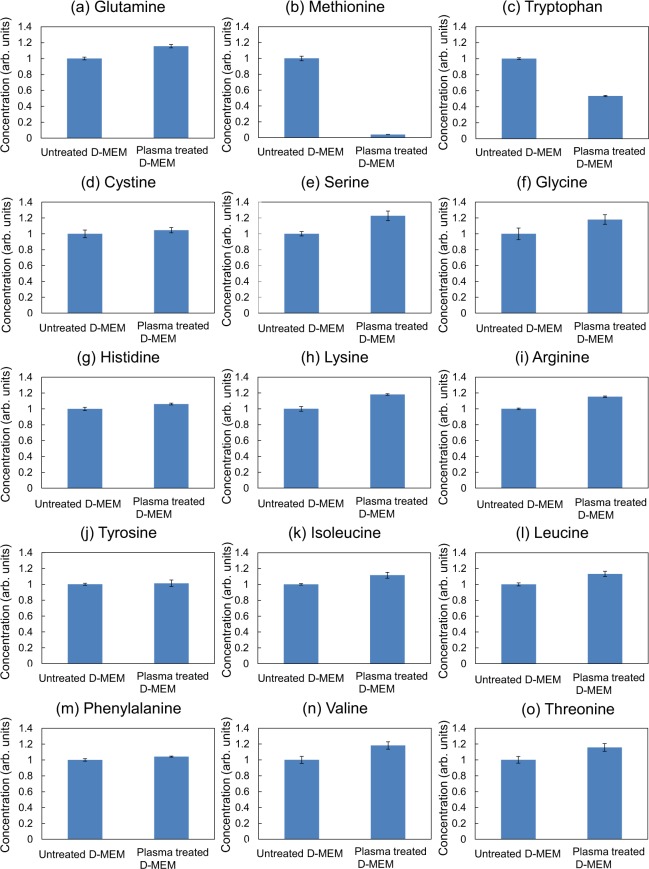
The concentrations of 15 amino acids in 9-min plasma-treated cell culture medium. The relative concentration of each amino acid after plasma irradiation to that in untreated cell culture medium is shown.

**Figure 2:**
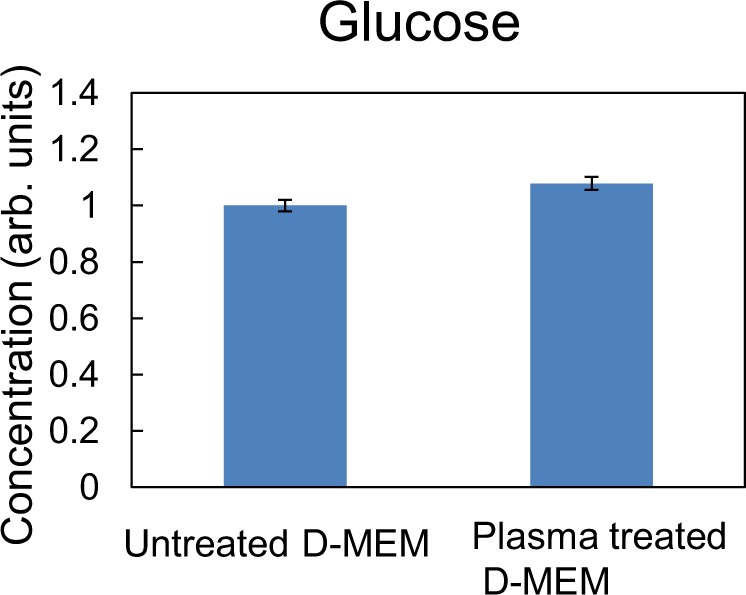
The concentration of glucose in 9-min plasma-treated cell culture medium. The relative concentration of glucose after plasma irradiation to that in untreated cell culture medium is shown.

First, a plasma jet was used to irradiate 3 ml of cell culture medium (WAKO, D-MEM 044-29765) without fetal bovine serum and penicillin streptomycin for 9 min and we investigated changes in the concentrations of 15 amino acids and glucose in the cell culture medium. The cell culture medium comprised 15 different amino acids, D-glucose, 8 vitamins, and 6 inorganic salts (CaCl_2_, KCl, MgSO_4_, NaCl, NaHCO_3_, and NaH_2_PO_4_). Figure 1 shows the amino acids concentrations before and after plasma treatment as measured by using an LC-MS/MS system. We observed a marked decrease in the concentration of (b) methionine and (c) tryptophan, suggesting that sulfur-containing and aromatic amino acids are preferentially destroyed by plasma irradiation, consistent with previous work reported by Takai *et al*.^33^. On the other hand, the concentration of (d) cystine, the other sulfur–containing amino acid, is not changed by plasma irradiation and rather is stable in plasma–treated medium. We also observed no significant change in glucose concentration before and after plasma treatment, as shown in Fig. 2. These results clearly show that plasma irradiation induces the oxidation and decomposition of specific amino acids such as methionine and tryptophan in cell culture medium.

**Figure 3:**
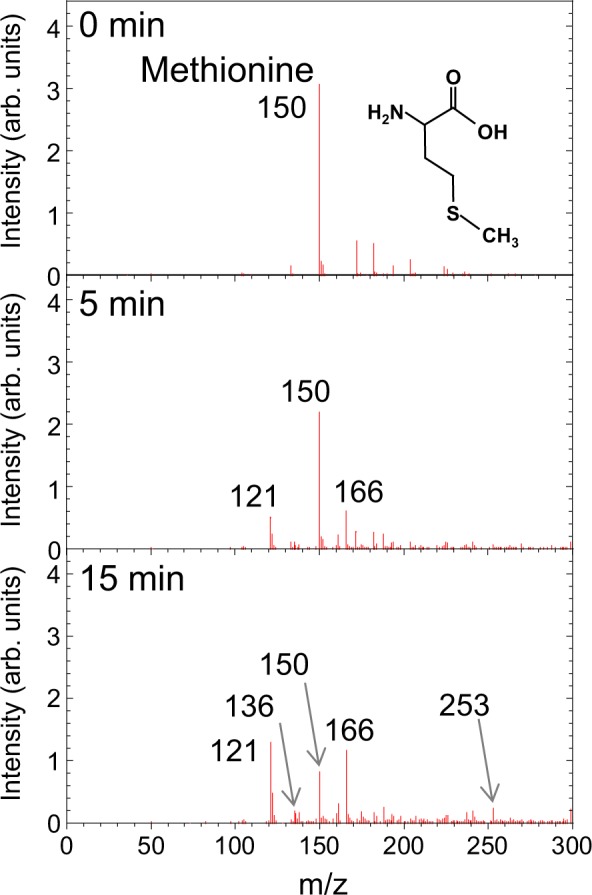
Mass spectra of methionine aqueous solution after plasma irradiation for 0, 5, and 15 min.

**Figure 4:**
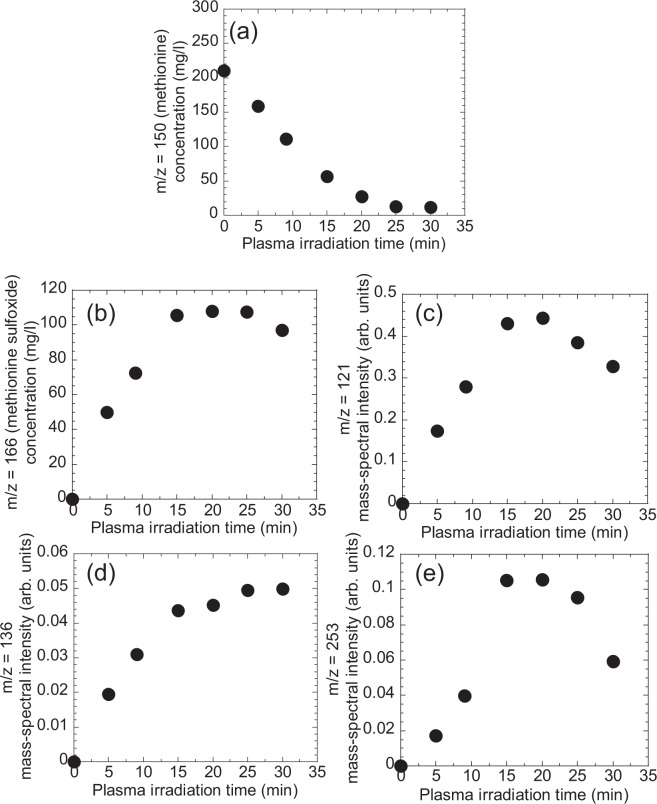
Plasma-irradiation time dependence of the mass-spectral intensity at m/z = (**a**) 150 (methionine), (**b**) 166 (methionine sulfoxide), (**c**) 121, (**d**) 136, and (**e**) 253 in plasma-treated methionine solution. The concentrations of methionine and methionine sulfoxide were evaluated from the mass-spectral intensity by comparison with a calibration curve of mass-spectral intensity vs. concentration.

Next, we analyzed in detail changes in methionine and tryptophan in cell culture medium upon plasma irradiation using electrospray ionization mass spectrometry by measuring the mass spectral m/z of plasma-treated deionized water containing either methionine or tryptophan. Here, m and z denote the mass and charge number of the observed molecule, respectively, and m is (amino acid + H). The volume of each sample was 3 ml and the concentration of amino acid was 210 mg/l. As shown in Fig. 3, there were marked changes in the mass spectrum of methionine at m/z = 150. After plasma irradiation for 5 and 15 min, new signals were observed, mainly at m/z = 121 and 166, which correspond to decomposition and oxidation products of methionine, respectively. In addition, many small signals were observed over a wide m/z range, for example m/z = 136 and 253. We could assign the oxidation product at m/z = 166 to methionine sulfoxide, which is produced by the oxidation of the sulfur in methionine (methionine + O). The decomposition products at m/z = 121 and 136 are tentatively assigned to (methionine − CH_2_ − NH) and (methionine − CH_2_), respectively. Figures 4(a)–(e) summarize the plasma-irradiation time dependence of the mass-spectral intensity at m/z = 150 (methionine), 166 (methionine sulfoxide), 121, 136, and 253, respectively. The concentration of methionine as determined by the peak intensity at m/z = 150 (methionine) decreased with plasma treatment up to 20 min while concomitantly the oxidation and decomposition products at m/z = 166 (methionine sulfoxide), 121, 136, and 253 increased. These results clearly show that plasma irradiation changes methionine chemically to products at m/z = 166 (methionine sulfoxide), 121, 136, and 253.

**Figure 5:**
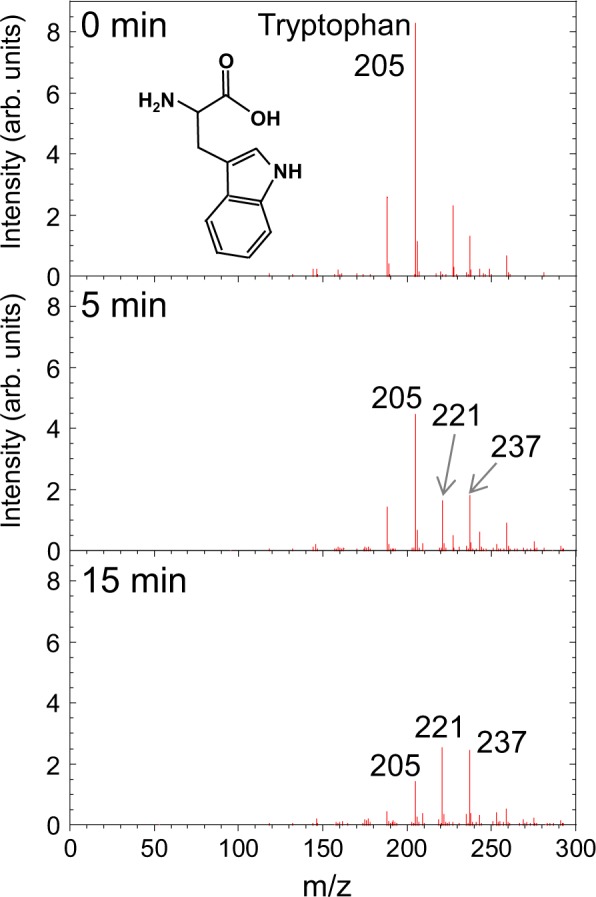
Mass spectra of tryptophan aqueous solution after plasma irradiation for 0, 5, and 15 min.

**Figure 6:**
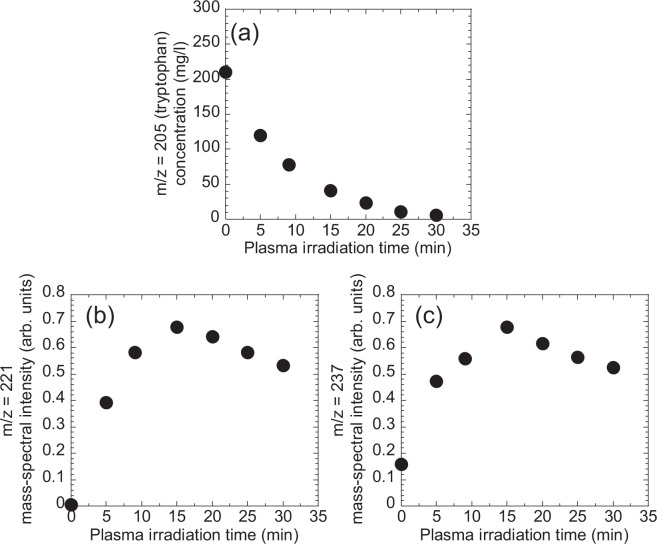
Plasma-irradiation time dependence of the mass-spectral intensity at m/z = (**a**) 205 (tryptophan), (**b**) 221, and (**c**) 237 in plasma-treated tryptophan solution. The concentration of tryptophan was evaluated from the mass-spectral intensity by comparison with a calibration curve of mass-spectral intensity vs. concentration.

Tryptophan gives a peak at m/z = 205 and plasma irradiation gave rise to distinct new signals at m/z = 221 and 237, corresponding to tryptophan oxidation products as shown in Fig. 5. We could assign the oxidation product at m/z = 221 to 5-hydroxy-L-tryptophan (tryptophan + O). Another oxidation product at m/z = 237 was tentatively assigned to (tryptophan + 2O) such as formylkynurenine, an intermediate in the catabolism of tryptophan^34^. Figures 6(a)–(c) show the plasma-irradiation time dependence of the mass-spectral intensity at m/z = 205 (tryptophan), 221, and 237, respectively. The concentration of tryptophan as estimated from the peak intensity at m/z = 205 (tryptophan) decreased with plasma irradiation up to 20 min while the oxidation products at m/z = 221 and 237 increased concurrently. These results clearly show that tryptophan chemically changed to products giving rise to peaks at m/z = 221 and 237 and explain the decrease in methionine and tryptophan in plasma-treated cell culture medium shown in Fig. 1.

**Figure 7:**
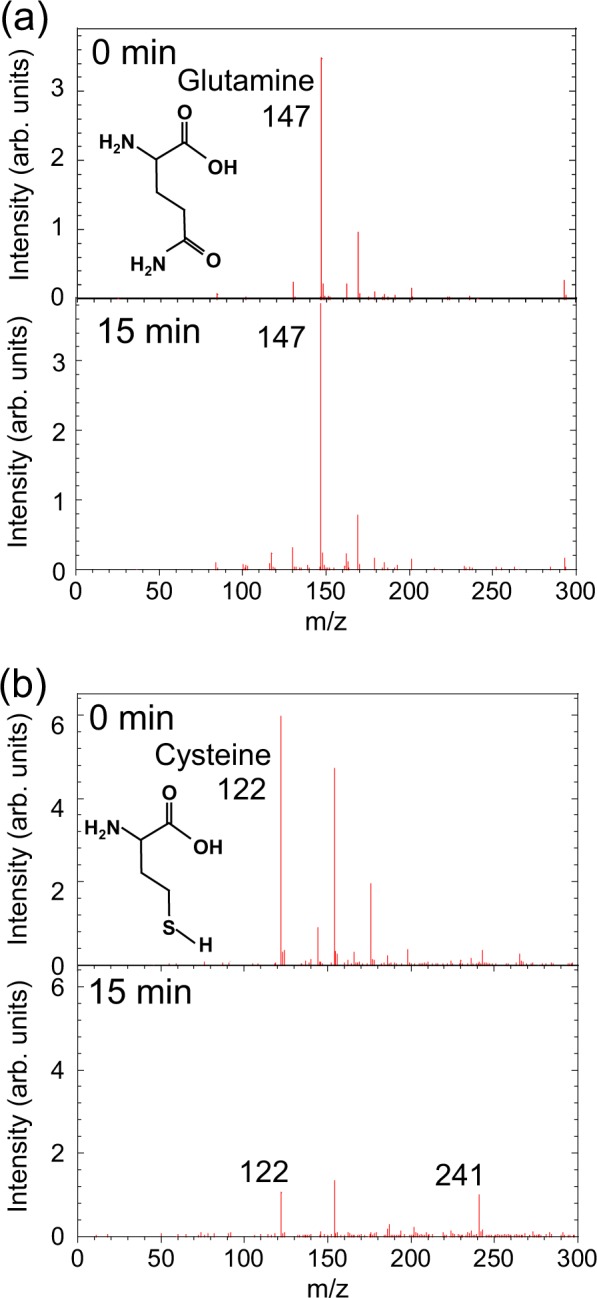
Mass spectra of (**a**) glutamine and (**b**) cysteine aqueous solution after plasma irradiation for 0 and 15 min.

We also analyzed changes in the mass spectrum of cysteine and glutamine, which have chemical structures similar to methionine. Glutamine lacks a sulfur group and cysteine is the other sulfur–containing amino acid (in addition to methionine), although cysteine is not present in cell culture medium. We found that glutamine (m/z = 147) is neither oxidized nor decomposed by plasma irradiation, as shown in Fig. 7(a). On the other hand, plasma irradiation for 15 min caused a decrease in the mass-spectral intensity of cysteine at m/z = 122 and a new signal corresponding to cystine at m/z = 241 appeared, as shown in Fig. 7(b). These results show that cysteine readily changed to the oxidized dimer form cystine upon plasma irradiation and the produced cystine was more stable than cysteine in plasma-treated solution. As shown in Fig. 1, the concentration of cystine did not change in cell culture medium after 9-min plasma irradiation but cystine might be oxidized by longer plasma irradiation, as reported previously^35^.

### ROS in plasma-treated amino-acid solutions

**Figure 8:**
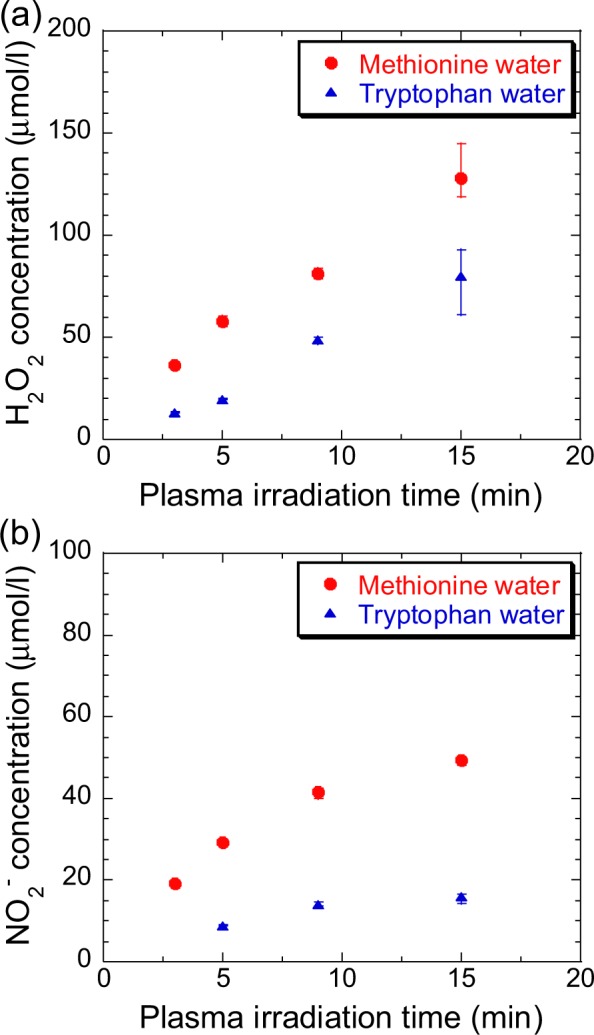
Dependence of (**a**) H_2_O_2_ and (**b**) NO_2_^−^ concentration in methionine and tryptophan solutions on plasma-irradiation time.

We measured H_2_O_2_ and NO_2_^−^ concentrations in sample solutions before and after plasma irradiation to understand the chemical reactions occurring in aqueous solutions of methionine and tryptophan during plasma irradiation. In particular, H_2_O_2_ is an important ROS with a long lifetime in water and reacts with methionine and tryptophan. The commercial chemical-probe reagents Amplex Red reagent (Thermo Fisher Scientific, Amplex Red Hydrogen Peroxide/Peroxidase Assay kit) and Griess reagent (DOJINDO, NO_2_/NO_3_ Assay Kit-C II) were used for the quantitative detection of H_2_O_2_ and NO_2_^−^, respectively. Figures 8(a) and (b) show the H_2_O_2_ and NO_2_^−^ concentrations, respectively, in plasma-treated methionine and tryptophan solutions as a function of plasma-irradiation time. The concentrations of both species increased with plasma-irradiation time, and approximately 100 μmol/l H_2_O_2_ was produced in methionine and tryptophan solutions during 15-min plasma irradiation.

**Figure 9:**
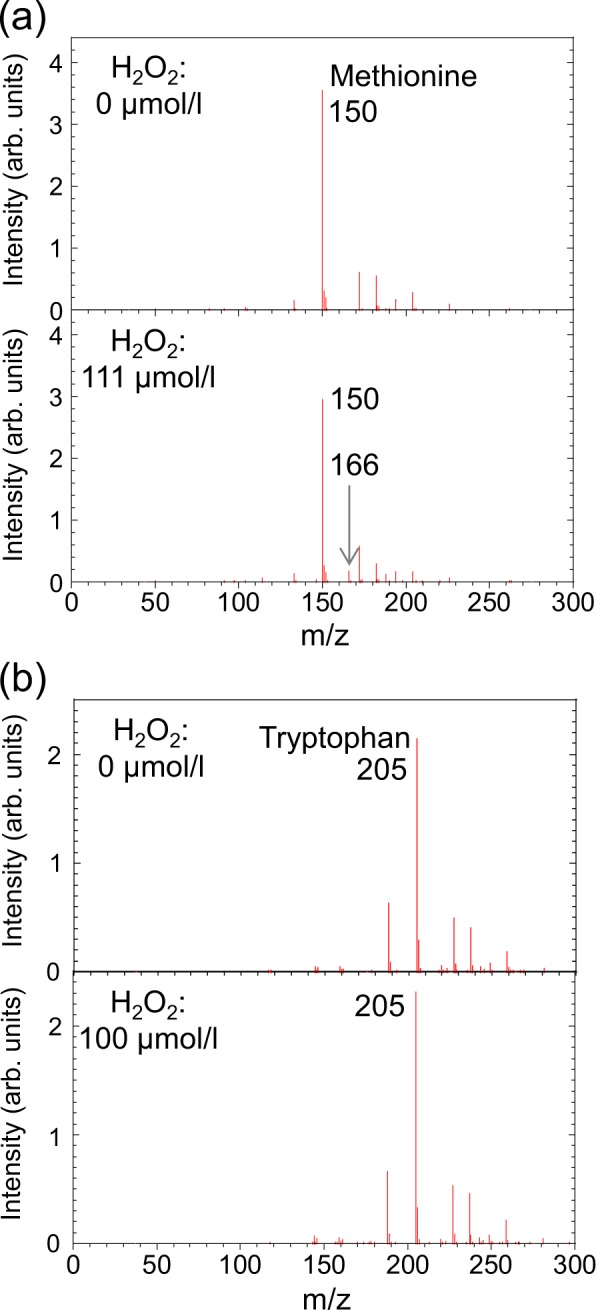
Mass spectra of (**a**) methionine and (**b**) tryptophan solution with/without exogenously added H_2_O_2_.

The effect of H_2_O_2_ on the oxidation and decomposition of methionine and tryptophan was determined by adding exogenous H_2_O_2_ to aqueous solutions of methionine or tryptophan. Figures 9(a) and (b) show the mass spectra of methionine and tryptophan solutions containing H_2_O_2_ concentrations of 0 and about 100 µmol/l, respectively. No significant changes were seen in the methionine signal at m/z = 150 and the tryptophan signal at m/z = 205, even at 100 µmol/l H_2_O_2_, indicating that H_2_O_2_ is ineffective for oxidizing and decomposing methionine and tryptophan.

**Figure 10:**
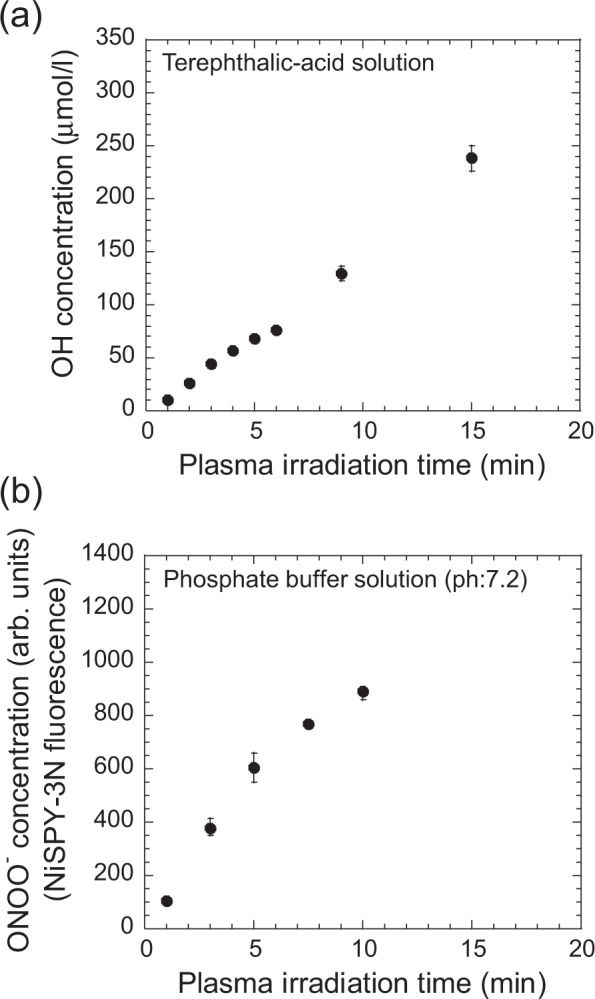
Dependence of (**a**) OH radical concentration in terephthalic-acid solution and (**b**) ONOO^−^ concentration (NiSPY-3N fluorescence) in phosphate buffer solution on plasma-irradiation time.

Plasma irradiation results in the abundant generation of many kinds of ROS in the irradiated solution. The OH radical has a short life time in water and is an important ROS for oxidation because of its high reaction rate constant with organic material (10^8^–10^9^ l/mol/s), which is much higher than that of H_2_O_2_ (10^5^–10^6 ^l/mol/s). The concentration of OH radicals was estimated by measuring the concentration of 2-hydroxyterephthalic acid and assuming that all the OH radicals in the solution are scavenged by terephthalic acid and converted to 2-hydroxyterephthalic acid. The plasma jet irradiated 3 ml of deionized water containing 4 mmol/l of terephthalic acid disodium salt. Figure 10(a) shows the plasma-irradiation time dependence of the OH radical concentration. The OH radical concentration linearly increased with plasma-irradiation time, consistent with the trend for H_2_O_2_ shown in Fig. 8(a). This is reasonable because OH radicals react with each other in solution to form H_2_O_2_ when the OH radical is not scavenged by terephthalic acid. We also measured ONOO^−^ in phosphate buffer solution (0.1 mol/l, pH = 7.2) by using the fluorescent reagent Nitrative Stress Sensing Pyrromethene Dye: NiPY-3 (Goryo Chemical, NiSPY-3). Compared to H_2_O_2_, ONOO^−^ has a 1000 times higher reaction rate constant with organic material containing a sulfhydryl group^36^. ONOO^−^ is relatively long lived at high pH but is rapidly protonated to form ONOOH in acid solution (pKa = 6.8), generating NO_3_^−^ and H^+^ ^10,37^. As shown in Fig. 10(b), the concentration of ONOO^−^ (NiPY-3N fluorescence) increased with plasma irradiation time. These results show that plasma-liquid interactions produce OH radical and ONOO^−^, species that might be effective for the oxidation and decomposition of amino acids.

### Anti-cancer effect of plasma-treated methionine and tryptophan solutions

**Figure 11:**
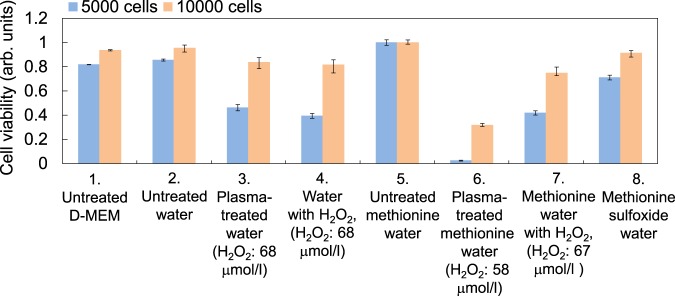
The viability of human endometrial carcinoma (HEC-1) cancer cells in plasma-treated methionine aqueous solution, evaluated using 5000 or 10000 cells. All samples were diluted by half with cell culture medium (D-MEM) without fetal bovine serum and penicillin streptomycin.

Finally, we investigated whether methionine and tryptophan solutions treated with plasma can kill cancer cells. This important biomedical application requires understanding the antitumor effects of plasma-treated cell culture medium reported previously^22^. Experiments on plasma-treated solutions containing only one cell culture medium component can help elucidate the mechanism of the antitumor effects. Figure 11 shows the viability of human endometrial carcinoma (HEC-1) cancer cells in the presence of plasma-treated water (sample No. 3) and plasma-treated methionine solution (sample No. 6) containing 5000 or 10000 cells (the number of cells added to the well before adding the plasma-treated solution). All samples were diluted to half the original concentration with cell culture medium (WAKO, D-MEM 044-29765) without fetal bovine serum and penicillin streptomycin and adjusted to the same salt concentrations (0.2 g/l CaCal_2_, 0.4 g/l KCl, and 6.4 g/l NaCl) by adding solid CaCal_2_, KCl, and NaCl to maintain osmotic pressure appropriate for the cells. It is worth noting that the pH of the plasma-treated solution samples was about 8.4 after diluting by half with cell culture medium, and the pH value was found to not affect cancer cell susceptibility in these experiments. We evaluated the cell viability after incubating HEC-1 cancer cells for about 24 hours in plasma-treated solution sample using cell proliferation assay reagent (TaKaRa, Premix WST-1)^26,30^. The plasma-treated water sample (sample No. 3) showed lower cell viability than both the untreated D-MEM sample (sample No. 1) and the untreated water sample (sample No. 2), likely due to H_2_O_2_ produced by plasma irradiation given that sample No. 3 showed the same cell viability as water containing the same concentration (68 µmol/l) of exogenously added H_2_O_2_ (sample No. 4). In contrast, the sample containing plasma-treated methionine solution (sample No. 6) showed much lower cell viability than both the sample containing methionine and exogenously added H_2_O_2_ (sample No. 7) and plasma-treated water (sample No. 3), when the 3 samples have almost the same H_2_O_2_ concentration. This clearly shows that the observed drastically reduced viability of cells exposed to plasma-treated methionine solution is not due to the presence of H_2_O_2_ and the depletion of methionine, an essential nutrient, but rather is caused by methionine oxidation or its decomposition products. We also found that solution containing 210 mg/l methionine sulfoxide (m/z = 166) (sample No. 8) had no effect on cell viability, despite the concentration of methionine sulfoxide being higher than its concentration in plasma-treated methionine aqueous solution, showing that methionine sulfoxide (methionine + O) produced by plasma irradiation is not toxic towards cancer cells.

**Figure 12:**
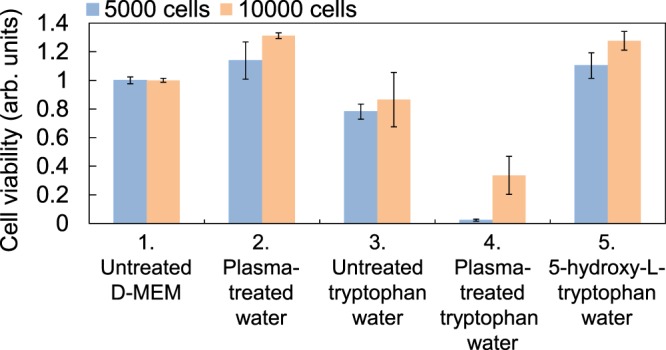
The viability of human endometrial carcinoma (HEC-1) cancer cells in plasma-treated tryptophan aqueous solution, evaluated using 5000 or 10000 cells. All samples were diluted by half with cell culture medium (D-MEM) without fetal bovine serum and penicillin streptomycin.

We examined the plasma-treated tryptophan solution in precisely the same manner. As shown in Fig. 12, the plasma-treated tryptophan solution sample (sample No. 4) showed much lower cell viability than the untreated D-MEM sample (sample No. 1), plasma-treated water sample (sample No. 2), and untreated tryptophan solution sample (sample No. 3). This shows that the observed drastically reduced viability of cells exposed to plasma-treated tryptophan solution is caused by tryptophan oxidation products. We also found that the solution containing 210 mg/l 5-hydroxy-L-tryptophan at m/z = 221 (sample No. 5) had no effect on cell viability, showing that 5-hydroxy-L-tryptophan (tryptophan + O) produced by plasma irradiation is not toxic towards cancer cells.

The cell viability observed in the plasma-treated methionine and tryptophan solution samples was much lower in the 5000-cell experiment than in the 10000-cell experiment, indicating that the amount of produced toxic material was insufficient to kill 10000 cells. We also confirmed that cell viability increased both in the 5000- and 10000-cell experiments when the dilution ratio of the plasma-treated methionine solution with D-MEM was decreased from 50% to 25%, likely due to a decrease in the concentration of the produced toxic material in the solution samples. The results show that the observed drastically reduced viability of cells is not due to the depletion of an essential nutrient such an amino acid, but rather is caused by toxic material produced in the plasma-treated solution samples.

**Figure 13:**
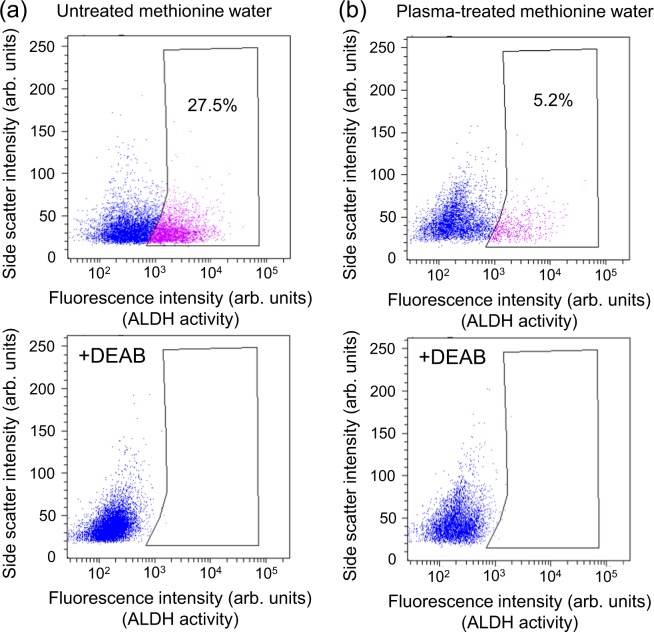
Effect of plasma-treated methionine solution on aldehyde dehydrogenase (ALDH) activity in human endometrial carcinoma (HEC-1) cancer cells. Top: Dot-blot data from Aldefluor assay of cells grown in the absence of the ALDH-specific inhibitor DEAB (diethylaminobenzaldehyde). Bottom: Dot-blot data for cells grown in the presence of the ALDH inhibitor DEAB. (**a**) Plasma-untreated methionine solution and (**b**) plasma-treated methionine solution. All samples were diluted by half with cell culture medium (D-MEM) without fetal bovine serum and penicillin streptomycin.

**Figure 14:**
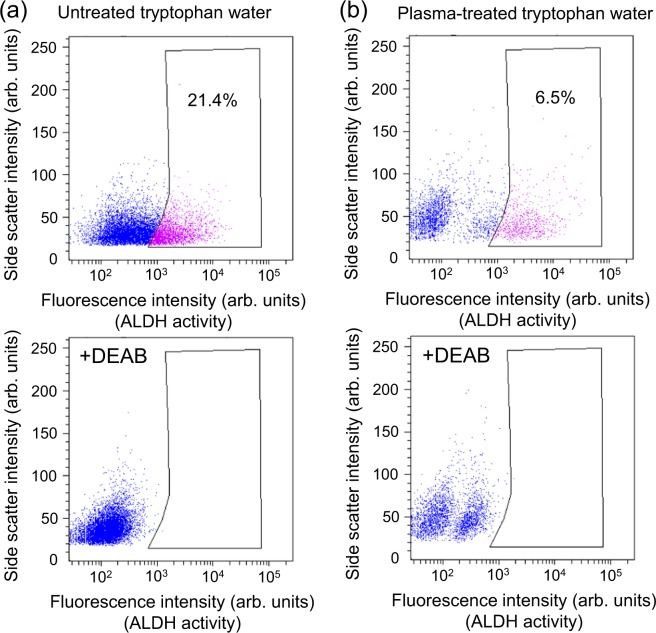
Effect of plasma-treated tryptophan solution on aldehyde dehydrogenase (ALDH) activity in human endometrial carcinoma (HEC-1) cancer cells. Top: Dot-blot data from Aldefluor assay of cells grown in the absence of the ALDH-specific inhibitor diethylaminobenzaldehyde (DEAB). Bottom: Dot-blot data for cells grown in the presence of the ALDH inhibitor DEAB. (**a**) Plasma-untreated tryptophan solution and (**b**) plasma-treated tryptophan solution. All samples were diluted by half with cell culture medium (D-MEM) without fetal bovine serum and penicillin streptomycin.

Furthermore, we analyzed the effect of plasma-treated methionine and tryptophan solutions on the viability of cancer-initiating cells. Such cells are proposed to cause recurrence or metastasis due to their resistance to anti-cancer drugs and radiation. Here, we evaluated aldehyde dehydrogenase (ALDH) activity as a cancer-initiating cell marker because cancer-initiating cells show high ALDH activity^38^. We examined changes in ALDH activity after incubating HEC-1 cancer cells for about 24 hours in plasma-treated methionine or tryptophan solution using an Aldefluor assay kit (StemCell Technologies Inc. Corp., ALDEFLUOR Kit)^26,30^. This assay involves measuring the fluorescence and light-side-scattering intensity of cells with a flow cytometer. HEC-1 cells with bright or faint fluorescence are defined as ALDH-high and ALDH-low activity cells, respectively, and are represented as pink and blue dots, respectively, as shown in the upper graphs in Figs. 13 and 14. ALDH-high activity cells (pink dots) correspond to cancer initiating-cells. Cell incubation in plasma-treated methionine solution markedly reduced the ALDH-high activity cells from 27.5 to 5.2%, as shown in Fig. 13(b) (upper panel), and incubation in plasma-treated tryptophan solution reduced ALDH-high activity cells from 21.4 to 6.5%, as shown in Fig. 14(b) (upper panel). The results suggest that plasma-treated methionine and tryptophan solutions induce an anti-cancer effect on cancer-initiating cells. We also confirmed that bright fluorescent cells were absent in the negative control, generated by adding the specific ALDH inhibitor diethylaminobenzaldehyde (DEAB), as shown in the lower graphs in Figs. 13 and 14.

## Discussion

**Figure 15:**
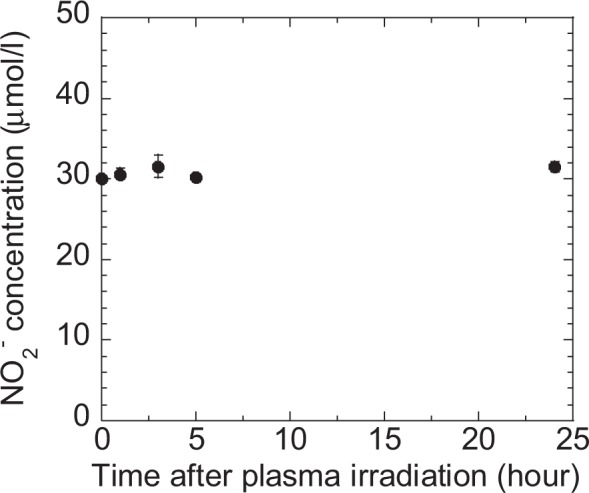
Dependence of NO_2_^−^ concentration in plasma-treated methionine solution on time after plasma-irradiation.

In this study we demonstrated that plasma-treated methionine and tryptophan solutions can kill HEC-1 cancer cells efficiently. ROS such as OH and NO that have short life times of less than a few minutes have no direct effect on cancer cells, given that the cells was placed in the plasma-treated solution at least 30 minutes after irradiation. However, if plasma treatment results in the attachment of a nitroso group to the sulfur in methionine, the resulting S-nitrosothiols could act as NO donors in solution long after plasma irradiation^39,40^. Hibbs *et al*. reported that NO is the toxic agent produced by macrophages and can kill tumor cells^41^. We confirmed the effect of NO by measuring the concentration of NO_2_^−^ in plasma-treated methionine solution for 24 hours after plasma treatment in the absence of cancer cells. In solution, NO reacts with O_2_ and H_2_O to form NO_2_^−^ and H^+^ ^42,43^. As shown in Fig. 15, the NO_2_^−^ concentration remained unchanged for 24 hours. Consequently, the very small amount of NO present after plasma treatment is apparently insufficient to kill cancer cells.

The mechanism underlying apoptosis in cancer cells has been previously investigated. Tanaka *et al*. reported that plasma-treated cell culture medium, which contains amino acids, down-regulates multiple cell survival and proliferation signaling pathways, including a PIK/AKT signaling pathway and a Ras/MAPK signal pathway^22,25^. Our results suggest that, in addition to ROS, plasma-treated amino acid solutions may contribute to the down-regulation of the survival signaling pathways. Alternatively, a different antitumor mechanism could be involved and thus further study is required to elucidate the mechanism of plasma-treated amino-acid solutions in the killing of cancer cells.

Our experiments showed that amino acid oxidation and decomposition products other than ROS are responsible for a strong antitumor effect. In addition, we conclusively showed that (1) plasma irradiation of methionine generates decomposition and oxidation products that provide signals at m/z = 121 and 166, respectively, and that methionine sulfoxide (methionine + O) at m/z = 166 is not toxic toward cancer cells, and (2) plasma irradiation of tryptophan generates oxidation products that provide signals at m/z = 221 and 237, and 5-hydroxy-L-tryptophan (tryptophan + O) at m/z = 221 is not toxic toward cancer cells. Although the key products responsible for killing cancer cell could not be identified in the current study, this may be the first demonstration that amino acid oxidation and degradation products have a strong antitumor effect.

## Methods

### Experimental plasma-jet system and preparation of plasma-treated solutions

**Figure 16:**
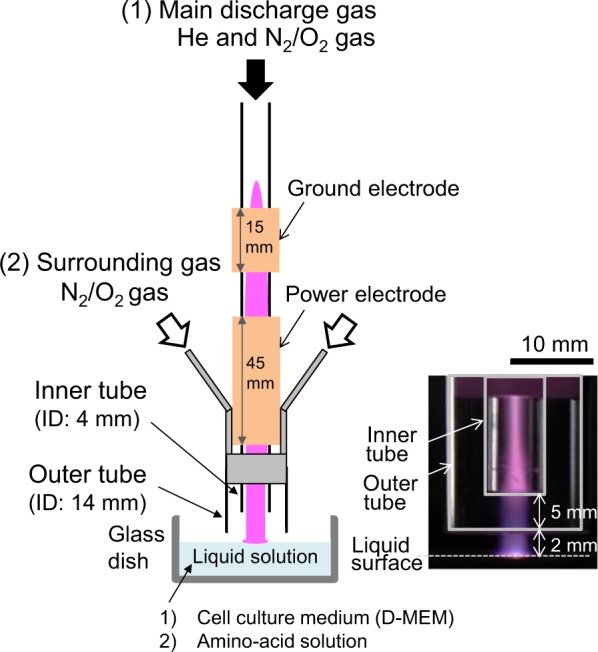
Experimental setup of the 5-kHz low-frequency plasma jet.

A low-frequency plasma jet was produced in the open air. A quartz tube was wrapped with 45- and 15-mm-wide copper metal strips as the power and ground electrodes, respectively, as shown in Fig. 16^44^. The outer and inner diameters of the quartz tube were 6 and 4 mm, respectively. The power electrode was set 30 mm away from the outlet of the quartz tube, and the distance between the power and ground electrodes was 8 mm. The power electrode was connected to a high positive rectangular voltage with an amplitude of 10 kV to initiate a dielectric barrier discharge between the power and ground electrodes in the quartz tube using a repetition frequency and duty ratio of the rectangular voltage of 5 kHz and 30%, respectively. We also set an outer quartz tube with an inner diameter of 14 mm outside the inner tube to introduce the surrounding gas near the plasma jet into the open air. The distance between the nozzles of the outer and inner quartz tubes was 5 mm. A gas mixture of He: 3 slm and N_2_(80%)O_2_(20%): 6 sccm was supplied to the inner quartz tube as the discharge gas, and a gas mixture of N_2_(80%)O_2_ (20%): 1 slm was supplied to the outer quartz tubes as the surrounding gas.

We used the plasma jet to irradiate 3 ml of cell culture medium (WAKO, D-MEM 044-29765) without fetal bovine serum and penicillin streptomycin, methionine solution, tryptophan solution, glutamine solution, and cysteine solution, all at a concentration of 210 mg/l, in a quartz cell with an inner-diameter of 34 mm and height of 17 mm or a 6-well plate. The distance between the outlet of the inner glass tube and the liquid surface was 7 mm.

### Measurement of the concentrations and mass spectra of amino acids

We analyzed changes in the concentration of amino acids and glucose in plasma-treated cell culture medium using an LC-MS/MS system (Shimadzu Corp., Nexera X2/LCMS-8050) as described previously with slight modifications^45^. The LC/MS/MS Method Package for Cell Culture Profiling (Shimadzu Corp.) provides optimized analytical conditions for up to 95 compounds, either as basal cell culture media components or as secreted metabolites, using LC-MS/MS data. In addition, changes in the mass spectrum of an amino acid in plasma-treated amino acid solution were analyzed with a liquid chromatography mass spectrometer (Shimazu Corp., LCMS-2020). The operating conditions of the electrospray ionization mass spectrometer (ESI-MS) were as follows: (1) electrospray potential = 4.50 kV, (2) capillary voltage = 0 V, (3) capillary temperature = 250 °C, (4) detector voltage = −1.30 kV, (5) skimmer voltage = 0 V, and (6) m/z scan range = 10–300, where m and z represent mass and charge number, respectively. The mass resolution of the LCMS-2020 as defined by m/Δm_50%_ is 2 m, where Δm_50%_ is the peak width at 50% of the maximum peak intensity^46^.

### Measurement of ROS in solution

We estimated the concentrations of H_2_O_2_ and NO_2_^−^ in plasma-treated solutions. These ROS are the two major reactive species with long lifetimes generated by plasma irradiation of aqueous solutions. H_2_O_2_ was detected using a molecular-probe assay kit (Thermo Fisher Scientific, Amplex Red Hydrogen Peroxide/Preoxidase Assay Kit). Amplex Red reacts with H_2_O_2_ in the presence of peroxidase to produce the red-fluorescent oxidation product resorufin, which has an absorbance maximum at approximately 550 nm. NO_2_^−^ was detected using another molecular-probe assay kit (DOJINDO, NO_2_/NO_3_ Assay Kit-C II) based on the Gries assay. The Gries assay uses the azo coupling between diazonium species produced from the reaction of sulfanilamide with NO_2_, together with naphthylethylenediamine, resulting in a colorimetric product with an absorbance maximum at approximately 550 nm. These commercial chemical-probe reagent kits are widely used to evaluate H_2_O_2_ and NO_2_^−^ in plasma-treated liquids^28,47–49^. After irradiating the sample solution with the plasma jet, the assay reagent was added to 50 μl of sample solution for H_2_O_2_ measurements and to 80 μl for NO_2_^−^ measurements in a 96-well plate and the absorbance was measured at 550-nm with a microplate photometer (Thermo Fisher Scientific, Multiskan FC). The concentrations of H_2_O_2_ and NO_2_^−^ were evaluated from the 550-nm absorbance by comparison with a calibration curve of absorbance vs. concentration.

We estimated OH radical concentrations in the solutions using terephthalic acid disodium salt (C_8_H_4_Na_2_O_4_)^50^. Terephthalic acid is an OH-radical scavenger that does not react with other ROS such as H_2_O_2_ and O_3_. The OH radical in solution converts terephthalic acid to 2-hydroxyterephthalic acid, which can be detected by fluorescence measurement. We irradiated the plasma jet onto 3 ml of deionized water containing 4 mmol/l of terephthalic acid disodium salt. The pH was not adjusted by adding buffer solution. The fluorescence of 150 μl of the plasma-treated sample in a 96-well plate was measured at 430 nm following excitation at 317 nm with a microplate photometer (Molecular Devices, Gemini XPS).

We also measured the amount of ONOO^−^ in the solutions using the fluorescent reagent Nitrative Stress Sensing Pyrromethene Dye: NiPY-3 (Goryo Chemical, NiSPY-3)^51–54^. NiSPY-3 reagent produces the fluorescence dye NiSPY-3N via nitration with ONOO^−^ and does not react with OH, O_2_^−^, H_2_O_2_, ^1^O_2_, OCl^−^, or NO. We irradiated the plasma jet onto 3 ml of sample solution containing 100 μmol/l of NiSPY-3. A stock solution was prepared by dissolving 1 mg NiSPY-3 powder in 430 μl of dimethyl sulfoxide, then diluting 100 times with 0.1 mol/l phosphate buffer solution. The pH of the NiSPY-3 solution was maintained at 7.2 by the phosphate buffer because ONOO^−^ has an acid dissociation constant (pKa) of 6.8 and is relatively stable in alkaline solution. After plasma irradiation, the fluorescence of 150 μl of the plasma-treated sample in a 96-well plate was measured at 520 nm following excitation at 480 nm with a microplate photometer (Molecular Devices, Gemini XPS).

### Assay of viability and ALDH activity of cells

**Figure 17:**
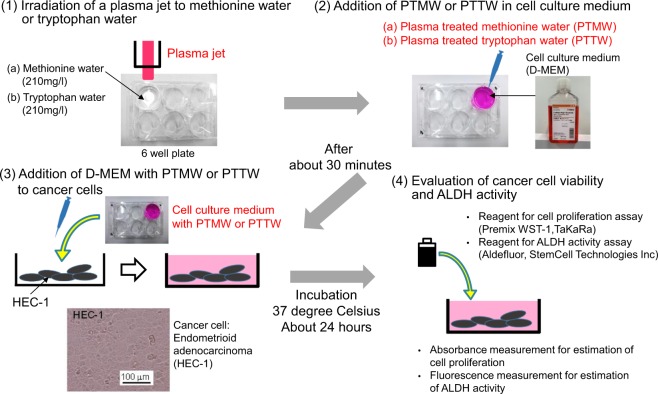
Procedure for evaluating the viability and aldehyde dehydrogenase (ALDH) activity of cancer cells in plasma-treated methionine and tryptophan solutions.

We evaluated the viability of cancer cells in plasma-treated methionine or tryptophan solution. A diagram of the experimental procedure is shown in Fig. 17. Sterilized water (WAKO, CultureSure 039-24155) was used to prepare all solutions. First, we irradiated a 210 mg/l methionine or tryptophan aqueous solution for 15 min, then diluted the sample by half with cell culture medium (WAKO, D-MEM 044-29765) without fetal bovine serum and penicillin streptomycin. Next, 200 µl of the original culture medium was removed and replaced with diluted plasma-treated methionine or tryptophan solution in a 96-well plate containing 5000 or 10000 human endometrial carcinoma (HEC-1) cancer cells. The plates were incubated in 5% CO_2_ at 37°C for about 24 hours, then cell viability was assayed by adding 20 μl of cell proliferation assay reagent (TaKaRa, Premix WST-1) and measuring the absorbance at 450 nm according to the manufacturer’s instructions^26,30^. The absorbance increases linearly with the number of viable cells and was measured with a microplate photometer (Corona, MTP-series). The Premix WST-l reagent enables measurement of cell viability using a colorimetric assay and is based on the cleavage of tetrazolium salts by mitochondrial dehydrogenase in viable cells. The number of viable cells is measured by detecting cleaved tetrazolium salts added to the cell medium.

We also evaluated aldehyde dehydrogenase (ALDH) activity in HEC-1 cancer cells to analyze the effect of plasma-treated methionine and tryptophan solutions on the viability of cancer-initiating cells. ALDH is a cancer-initiating cell marker in many types of tumors because cancer-initiating cells show high ALDH activity^38^. We examined and isolated the cell population exhibiting high ALDH activity using an Aldefluor kit (StemCell Technologies Inc. Corp.) according to the manufacturer’s instructions and previous reports^26,30^. Briefly, cells were suspended in Aldenuor assay buffer containing ALDH substrate and BODIPY-aminoacetaldehyde (BAAA). BAAA is taken up by live cells and converted by intracellular ALDH into BODIPY-aminoacetate, which yields bright fluorescence. The brightly fluorescent ALDH expressing cells were detected with a flow cytometer (BD Bioscience, FACS Canto II). Data were analyzed using Cell quest software (BD Bioscience). As a negative control, cells were stained under identical conditions in the presence of the specific ALDH inhibitor diethylaminobenzaldehyde (DEAB). We removed 3 ml of the original culture medium and replaced it with diluted plasma-treated methionine or tryptophan solution in a 6-well plate containing 150000 HEC-1 cancer cells, then incubating in 5% CO_2_ at 37°C for about 24 hours. ALDH activity was assayed using the above-mentioned Aldefluor kit and flow cytometer. Cells with bright or faint fluorescence were distinguished as ALDH-high and ALDH-low activity cells, respectively. Cells with high ALDH activity correspond to cancer-initiating cells.

The HEC-1 cancer cells were obtained from the Health Science Research Resource Bank of Osaka, Japan, and were cultured in Dulbeco’s modified Eagle’s medium (DMEM) supplemented with 10% fetal bovine serum and penicillin streptomycin.
