# Publisher Correction: Decomposition and oxidation of methionine and tryptophan following irradiation with a nonequilibrium plasma jet and applications for killing cancer cells

**DOI:** 10.1038/s41598-019-54737-3

**Published:** 2019-11-27

**Authors:** Giichiro Uchida, Yusuke Mino, Tensho Suzuki, Jun-ichiro Ikeda, Takashi Suzuki, Kosuke Takenaka, Yuichi Setsuhara

**Affiliations:** 1 Joining and Welding Research Institute, Osaka University, Ibaraki, Osaka, 567-0047 Japan; 2 Graduate School of Medicine, Osaka University, Suita, Osaka, 565-0871 Japan; 3 Analytical and Measuring Instruments Division, Shimadzu Corporation, Kyoto, Kyoto 604-8511 Japan; 4 Present Address: Graduate School of Medicine, Chiba University, Chiba, 260-8670 Japan

Correction to: *Scientific Reports* 10.1038/s41598-019-42959-4, published online 29 April 2019

In Figure 5 there is a missing axis label. The y-axis of the bottom graph should be labelled “Intensity (arb. units)” The correct Figure 5 appears below as Figure 1.

**Figure 1:**
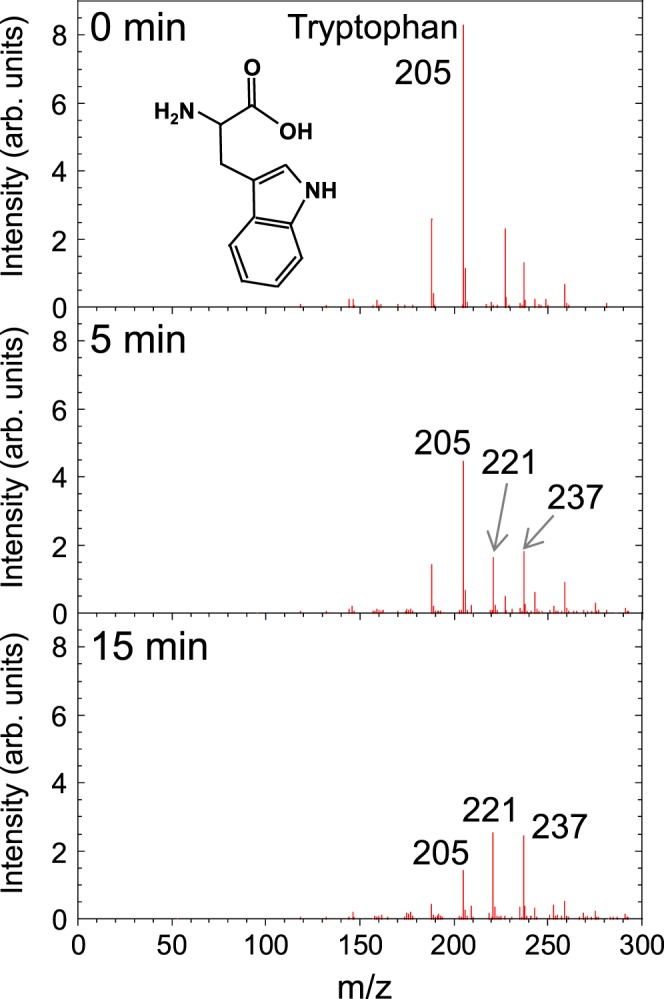
.

Additionally, this Article contains errors in Reference 44 which is incorrectly given as:

Ito, T., Uchida, G., Nakajima, A., Takenaka, K. & Setsuhara, Y. Selective production of reactive oxygen and nitrogen species in the plasma-treated water by using a nonthermal high-frequency plasma jet. *Jpn. J. Appl. Phys*. **56**, 01AC06 (2016)

The correct reference is listed below as ref. 1:
